# Influence of Coarse Aggregates and Silica Fume on the Mechanical Properties, Durability, and Microstructure of Concrete

**DOI:** 10.3390/ma12203324

**Published:** 2019-10-12

**Authors:** Seong Soo Kim, Abdul Qudoos, Sadam Hussain Jakhrani, Jeong Bae Lee, Hong Gi Kim

**Affiliations:** 1 Department of Civil Engineering, Daejin University, Pocheon-si 11159, Korea; sskim@daejin.ac.kr; 2 Civil Engineering Department, Balochistan University of Engineering and Technology, Khuzdar 89100, Pakistan; qudoos.engnr@gmail.com; 3 Department of Civil and Environmental Engineering, Hanyang University, Seoul 04763, Korea; sadamhussain@hanyang.ac.kr; 4 GFC R&D CO., Ltd., Pocheon-si 11159, Korea; dlwjdqo@nate.com

**Keywords:** concrete, aggregates, mechanical properties, durability

## Abstract

Globally, concrete is the most widely used construction material. The composition of concrete plays an important role in controlling its overall performance. Concrete is composed of approximately 70%–80% aggregates, by volume. Therefore, it is mandatory to investigate the effect of aggregates on the performance of concrete. For this purpose, this study investigated the effect of three different coarse aggregates on the mechanical properties, durability, and microstructure of concrete. Concrete specimens were made using aggregates obtained from three regions with different mineralogies. The specimens were also made by replacing cement with silica fume. The specimens were analyzed in terms of compressive, flexural, and splitting tensile strengths, chloride penetration, carbonation, mercury intrusion porosimetry, and scanning electron microscopy. The results demonstrate that the specimens made with rougher coarse aggregates and silica fume had enhanced performance in comparison to those made with smoother aggregates.

## 1. Introduction

Concrete is the most widely used construction material in the world. Around 10 billion tons of concrete are produced annually worldwide [1]. This amount is expected to increase to about 18 billion tons per year by 2050 [2]. The composition of concrete plays a vital role in controlling the overall performance of the cement composites. The binder is the main ingredient of the concrete composition, which has a significant impact on the performance of cement composites. Currently, the binder is modified to obtain high-strength, high-performance concrete. For this purpose, supplementary cementitious materials (SCMs) are used to replace a portion of the cement. The most commonly used SCMs are silica fume, ground granulated blast furnace slag (GGBS), fly ash, and metakaolin. These materials contain significant amounts of amorphous silica, which reacts with calcium hydroxide, a hydration product of cement, and forms additional calcium silicate hydrate gel. Current literature on the use of silica fume suggests that the addition of silica fume results in enhanced strength of the cement composites [3]. In addition, durability-related properties like sulfate and freeze–thaw resistance and electrical resistivity were improved, while sorptivity, water absorption, and chloride penetration were reduced due to the addition of silica fume [4,5]. Similarly, the addition of these SCMs significantly enhances the microstructure of the interfacial transition zone (ITZ), the relatively weak zone between the aggregate and the cement paste in concrete [6]. The type of aggregate also contributed to the performance of cement composites.

Aggregates occupy approximately 70% of the total volume of concrete. Therefore, the properties of aggregates play an important role in the overall performance of cementitious composites [7]. Ahmad and Alghamdi [8] investigated the effect of two types of coarse aggregates (i.e., calcareous limestone and basalt on compressive strength, modulus of elasticity, and steel corrosion resistance of concrete). They made concrete specimens by varying water:binder ratio, fine:total aggregate ratio, and cementitious materials content. They concluded that the quality of coarse aggregate affected both the mechanical properties and corrosion resistance of concrete. Concrete specimens prepared with limestone (abrasion: 25%) presented better performance than basalt (abrasion: 37%) in terms of compressive strength and corrosion resistance. At higher water:cementitious ratio, the effect of aggregate quality was lower than that at lower water:cementitious ratio. Beshr et al. [9] investigated the influence of four types of coarse aggregates on compressive and tensile strengths, and elastic modulus of high-strength concrete. They used calcareous, dolomitic, quarzitic limestone, and steel slag aggregate. The results of their study demonstrate that the quality of coarse aggregates significantly affects the compressive strength of high-strength concrete. Highest compressive strength was presented by the concrete specimens made with steel slag aggregates, while that of the least compressive strength was observed for calcareous limestone. Similar behavior was observed for tensile strength and elastic modulus results. Özturan and Çeçen [10] evaluated the effect of three types of coarse aggregates (i.e., basalt, limestone, and gravel) on the compressive, flexural, and split tensile strengths of concrete with varying strength grades. The results of their study demonstrate that high-strength concrete specimens made with basalt and limestone coarse aggregates presented 10% to 30% higher compressive, flexural, and split tensile strengths in comparison with that made of gravel aggregates. However, least variation in strength results were observed for normal strength concrete specimens made with different aggregates. In addition, they reported that split tensile strength of high-strength concrete is mostly governed by the strength of the mortar, whereas compressive strength is significantly influenced by the strength and surface characteristics of coarse aggregates. 

It is evident that coarse aggregates play an important role in controlling the overall performance of concrete. In addition, the microstructure of interfacial transition zone (ITZ) is significantly affected due to the surface characteristics and quality of coarse aggregates [6]. It can be concluded from the literature review presented in a previous study [6] of the authors that ITZ plays an important role in controlling the mechanical as well as durability-related properties of concrete. Therefore, it is mandatory to investigate the influence of different types of aggregates on the mechanical properties, durability, and microstructure of concrete.

This study investigates the influence of different aggregate types and the addition of silica fume on the mechanical properties, durability, and microstructure of concrete. For this purpose, concrete specimens were made using aggregates obtained from three regions with different mineralogies and silica fume as cement replacement material. The specimens were analyzed in terms of compressive strength, ultrasonic pulse velocity, chloride penetration, carbonation, freeze–thaw resistance, and scanning electron microscopy.

## 2. Materials and Methods

### 2.1. Materials

Ordinary Portland cement (OPC) in compliance with ASTM C150 [11] was used as the main binder. Silica fume was used to replace a portion of the cement. The physical and chemical properties of cement and silica fume are presented in Table 1. A polycarboxylate-based superplasticizer was used to improve the durability of the concrete mixes. Natural sand with maximum size, fineness modulus (F.M.), and absorption of 5 mm, 2.7, and 1.07%, respectively, was used as a fine aggregate. Three types of coarse aggregates were used in this study, each of different mineralogical origin: granite (G), quartzite (Q), and basalt (B). Granite and basalt coarse aggregates were obtained from the Ahn-sung and Hwa-sung areas of Gyeong-gi province of South Korea. Quartzite was obtained from the Ok-chun area of Chung-buk province of South Korea. This study is an extension of the previous study of the authors [6]. Based on the results of that study, the type of aggregates were selected. The physical properties and chemical composition of these aggregates are summarized in Table 2 and Table 3, respectively.

### 2.2. Mix Proportions and Fabrication of Specimens

The basic mix proportions used to make concrete specimens were 450 kg/m^3^ of cement, 675 kg/m^3^ of fine aggregate, and 1125 kg/m^3^ of coarse aggregate. A water:binder ratio of 0.4 was used for all mixes. Silica fume was used to replace cement at a dosage level of 10% by weight of cement. The specimens made with only cement as binder were represented with the acronyms CG, CQ, and CB, while those containing silica fume were named SFG, SFQ, and SFB. Initially, the ingredients were dry-mixed in a laboratory-scale concrete mixer; then water was added. After mixing, concrete specimens were cast and placed at room temperature for 24 h. Concrete cylinders (ϕ100 × 200 mm^3^) and concrete beams (100 × 100 × 400 mm^3^) were made. The molds were sealed with plastic sheets to avoid water loss due to evaporation. After 24 h, the specimens were removed from the molds and placed in water until the testing age was reached. At the desired test age, three replicates for each mix were used for investigating the desired parameters. 

### 2.3. Testing Methods

#### 2.3.1. Compressive Strength Test

A compressive strength test was carried out on the cylindrical specimens with dimensions ϕ100 × 200 cm^3^ following ASTM C 39 [12]. Three replicates were used for each mix. A universal testing machine (CCM-200A; Shimadzu Corporation, Kyoto, Japan) was used for this test.

#### 2.3.2. Ultrasonic Pulse Velocity Test

Ultrasonic pulse velocity is an effective non-destructive testing method used to estimate the internal structure of the specimens. This test uses pulse waves, which are transmitted from one end and received at the other end of the specimen with the help of transducers. This test is applied to investigate the quality of the specimen in terms of the presence of voids and cracks [13]. This test was conducted on the concrete beams (100 × 100 × 400 mm^3^) following ASTM C597 [13]. Three replicates were used for each mix. The pulse velocity was calculated using the equation

V = L/T,(1)

where V—pulse velocity, m/s; L—distance between centers of the transducer faces, m; T—transit time, s.

#### 2.3.3. Chloride Penetration Test

**Figure 1:**
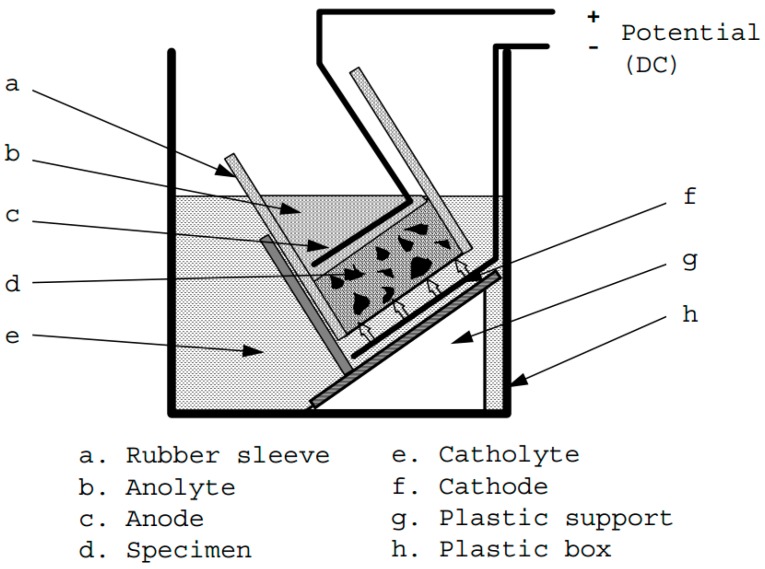
Test setup for chloride migration test [14].

The specimens for the chloride migration test were obtained following the procedure described in NT BUILD 492 [14]. The specimens were pre-conditioned by being immersed in a lime-rich solution for a period of 24 h. Then, the specimens were fitted in rubber sleeves and secured with two clamps in order to prevent leakage. The specimen surfaces were exposed to catholyte (10% NaCl) and anolyte (0.3 N NaOH) solutions, as shown in Figure 1. The test was initiated for a specific voltage adjustment and an appropriate duration. The specimens were split in half and sprayed with 0.1 M silver nitrate solution to measure the depth of chloride penetration. The non-steady-state migration coefficients of the specimens were calculated using the equation

(2) $$\mathrm{Dnssm}=\frac{0.0239\left(273+T\right)L}{\left(U-2\right)t}\left(x_{d}-0.0238\sqrt{\frac{\left(273+T\right)Lx_{d}}{U-2}}\right),$$

where D_nssm_—non-steady-state migration coefficient, ×10^−12^ m^2^/s; U—absolute value of the applied voltage, V; T—average value of the initial and final temperatures in the anolyte solution, °C; L—thickness of the specimen, mm; $x_{d}$—average penetration depth, mm; t—test duration, h.

#### 2.3.4. Carbonation Test

The concrete specimens of dimensions ф 100 × 50 mm were obtained from the cylinder specimens and placed in an accelerated carbonation chamber at 23 °C, 65% relative humidity, and 5% CO_2_ concentration. At the desired age, the specimens were removed from the chamber, split in half, and sprayed with 1% phenolphthalein solution in order to measure the depth of carbonation. A total of ten depth measurements were made for each specimen, and the average value is reported.

#### 2.3.5. Freeze–Thaw Resistance

The resistance of concrete to freezing and thawing was measured following ASTM C 666A [15]. After curing the concrete specimens in water for a period of 14 days, the specimens were placed in a freeze–thaw chamber (Model# HS-FTC660A, Hwasung, Incheon, South Korea, temperature range: −18 °C to 4 °C) and investigated for 300 cycles. Before starting the cycles, the length, cross-section, mass, and longitudinal transverse frequency of the specimens were noted. After every 30 cycles, the specimens were removed from the apparatus under thawing conditions and the parameters listed above were measured. Durability factor DF was calculated as:

DF = PN/M,(3)

where DF—durability factor of the test specimen; P—relative dynamic modulus of elasticity at N cycles, %; N—number of cycles at which P reaches the specified minimum value for discontinuing the test or the specified number of cycles at which the exposure is to be terminated, whichever is less; M—specified number of cycles at which the exposure is to be terminated.

#### 2.3.6. Scanning Electron Microscopy

The samples for scanning electron microscopy (SEM) and Energy-dispersive electron microscopy (EDS) analysis were obtained from the portions of concrete cylinders used for the compressive strength test [16]. A scanning electron microscope (Hitachi, Tokyo, Japan, accelerating voltage of 0.2–30 kV, probe current of 10E-12 to 10E-5A, SEI resolution of 3.5 nm (W.D = 8 mm, Acc.V = 35 kV), and magnification of 10× to 300,000×) was used to analyze the microstructural properties of the concrete. The samples were obtained and prepared for SEM analysis using the methods described in [16]. In this study, EDS elemental mapping (Hitachi, Tokyo, Japan, detector type of SDD Apollo XL, resolution of 134.49, take-off angle of 35) with an accelerating voltage of 20 kV was carried out along the aggregate surface to examine the distribution of elements. 

## 3. Results and Discussion

### 3.1. Compressive Strength

**Figure 2:**
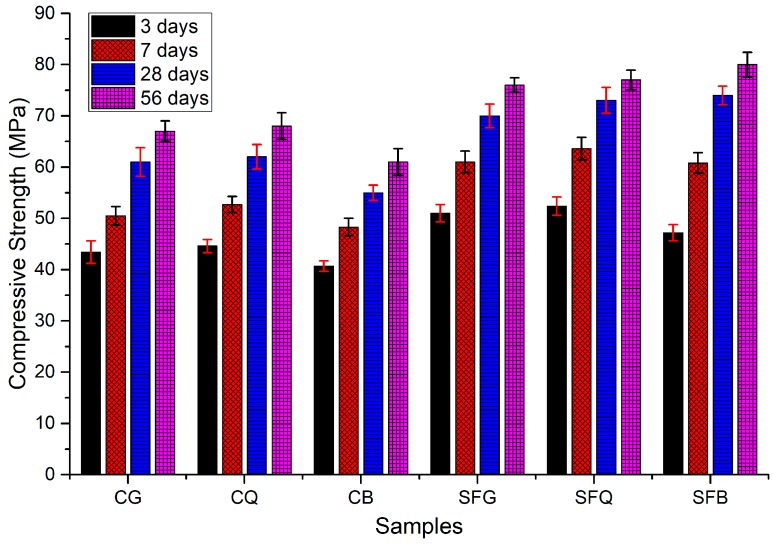
Compressive strength test results.

Compressive strength tests were conducted on the concrete specimens after 3, 7, 28, and 56 days of curing in water, as shown in Figure 2. The figure demonstrates that compressive strength increased with the increase in curing duration. The specimens made with coarse basalt aggregates had the least compressive strength. For instance, CB specimens demonstrated compressive strengths of 41, 48, 55, and 61 MPa at the ages of 3, 7, 28, and 56 days of curing in water, respectively. On the other hand, the specimens made with granite and quartzite presented enhanced compressive strength compared to those made with basalt. For example, at the ages of 3, 7, 28, and 56 days, compressive strengths of 45, 53, 62, and 68 MPa were measured for CQ concrete specimens, respectively. In a similar fashion, CG concrete specimens showed compressive strengths of 43, 51, 61, and 67 MPa at the ages of 3, 7, 28, and 56 days of curing. As discussed in a previous study conducted by the authors [6], aggregates with a rougher surface provide better packing of the cement particles, which ultimately result in an improved compressive strength. Similarly, the results of another study demonstrate that surface texture and abrasion value of the aggregates played an important role in governing the mechanical properties of the concrete specimens [17].

Chiaia et al. [18], Prokopski and Halbiniak [19], and Szczesniak et al. [20] reported that the stress at which cracks are produced mainly depends on the characteristics of the coarse aggregate. They concluded that aggregates with a relatively smooth, rounded surface inhibit crack propagation and initiation slightly. In a similar way, the results of previous studies [8,9,10] demonstrate that the quality and surface characteristics of coarse aggregates significantly influence the strength properties of concrete.

The incorporation of silica fume caused an increase in the compressive strength of the specimens, irrespective of the type of the aggregate. For instance, compressive strengths of 47, 61, 74, and 80 MPa were observed for the specimens made with basalt aggregate and silica fume at 3, 7, 28, and 56 days of curing, respectively. On the other hand, SFQ and SFG specimens demonstrated compressive strengths of 52 and 51 MPa at 3 days; 64 and 61 MPa at 7 days; 73 and 70 MPa at 28 days; 77 and 76 MPa at 56 days of curing, respectively. Interestingly, the specimens made with basalt aggregate and silica fume had improved compressive strength compared to those made with granite and quartzite aggregates and silica fume. This can be attributed to the greater strength of the basalt aggregate compared to those of the other aggregates. The addition of silica fume improved the interfacial transition zone; it is due to this reason the strength of the aggregate governed the mechanical properties of concrete specimens for this case. The results of the study conducted by Kilic et al. [17] indicate that the compressive strength of the concrete specimens increased with the increase in compressive strength of the parent rock of the aggregates. 

Ultimately, the addition of silica fume enhanced the compressive strength due to its highly pozzolanic behavior; the weaker aggregates presented weak planes during compression testing [21]. In addition, the fine particles of silica fume improved the microstructure near the aggregate surface due to filling and pozzolanic effects [6].

### 3.2. Ultrasonic Pulse Velocity

**Figure 3:**
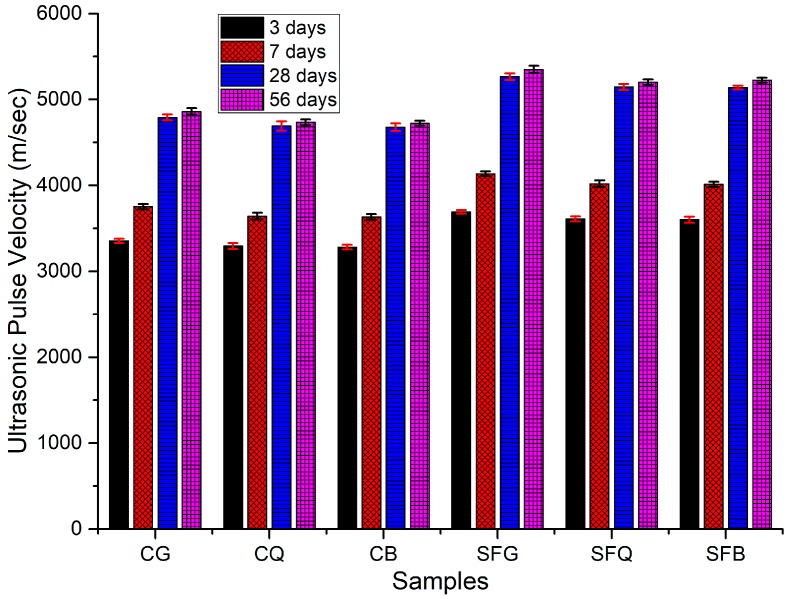
Ultrasonic pulse velocity test results.

In order to examine the microstructure of the concrete specimens, an ultrasonic pulse velocity test was conducted, and the results are reported in Figure 3. Similar to the results of the compressive strength test, pulse velocity increased with the increase in curing duration. However, the specimens made with basalt coarse aggregate presented comparable results to those made with granite and quartzite aggregates. For instance, the pulse velocity values for CB specimens were 3281, 3632, 4677, and 4723 m/s after 3, 7, 28, and 56 days of curing, respectively. The specimens made with quartzite demonstrated UPV values of 3295, 3640, 4690, and 4733 m/s after 3, 7, 28, and 56 days of curing, respectively. In the same way, after 3, 7, 28, and 56 days of curing, the specimens made with granite coarse aggregate demonstrated UPV values of 3354, 3752, 4790, and 4860 m/s, respectively. The higher UPV values for the CB specimens could be caused by the greater strength of the basalt aggregates, which have more closely packed grains compared to the granite and quartzite aggregates. Nevertheless, the packing of the cement particles around the basalt aggregate surface was not sufficient; the dense microstructure of the aggregate itself was responsible for higher UPV values.

The specimens made with silica fume depicted higher UPV values compared to those made without silica fume. For example, UPV values of 3601, 4012, 5138, and 5225 m/s were observed for the concrete specimens made with silica fume and basalt aggregate after 3, 7, 28, and 56 days of curing, respectively. In a similar fashion, SFQ concrete specimens showed UPV values of 3610, 4020, 5145, and 5200 m/s after 3, 7, 28, and 56 days, respectively. After 3, 7, 28, and 56 days of curing, UPV values of 3692, 4135, 5265, and 5350 m/s, respectively, were measured for SFG concrete specimens. Silica fume, being a highly pozzolanic material, consumes calcium hydroxide, resulting in the formation of calcium silicate hydrate. The formation of calcium silicate hydrate improves the microstructure of the cement composites, causing higher UPV values. Gesoglu and Guneyisi made rubberized concretes with silica fume as partial replacement for cement. The results of their study demonstrate that UPV values increased with the addition of silica fume [21]. 

### 3.3. Chloride Penetration, Carbonation, and Freeze–Thaw Resistance

**Figure 4:**
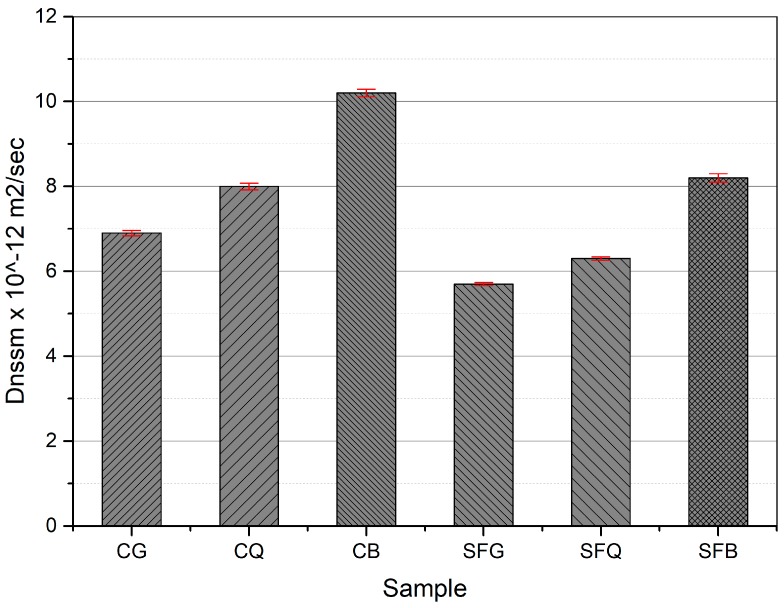
Non-steady-state migration coefficient values.

The concrete specimens were exposed to accelerated chloride and carbonation environments, and results are shown in Figure 4. It is clear from the results that the specimens made with basalt aggregate presented higher D_nssm_ values compared to those made with granite or quartzite aggregates. For example, the specimens made with basalt, quartzite, and granite aggregates demonstrated D_nssm_ values of 10.2 × 10^−12^, 8 × 10^−12^, and 6.9 × 10^−12^ m^2^/sec, respectively. These results followed a linear trend with a R^2^ value of 0.96. On the other hand, the inclusion of silica fume particles reduced the D_nssm_ values of all the specimens. For instance, D_nssm_ values of 8.2 × 10^−12^, 6.3 × 10^−12^, and 5.7 × 10^−12^ m^2^/sec were measured for SFB, SFQ, and SFG concrete specimens, respectively. A linear trend with R^2^ value of 0.91 was observed for these results. 

**Figure 5:**
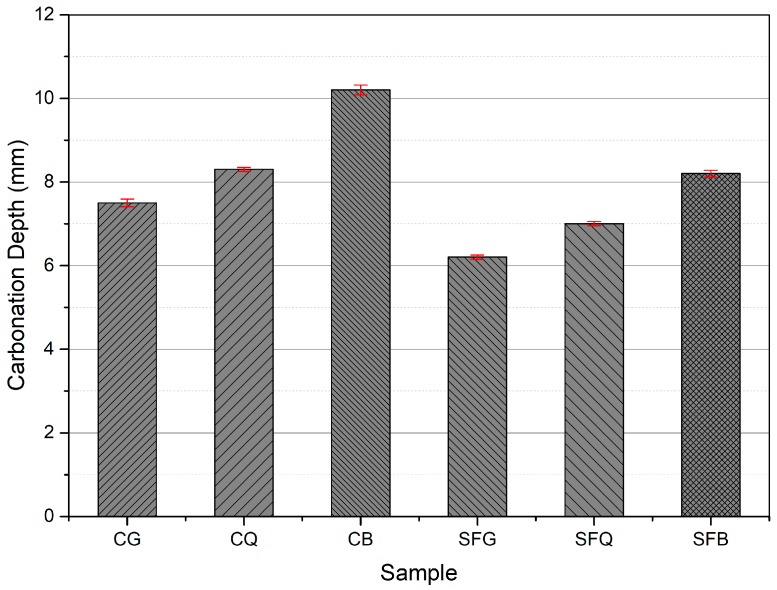
Carbonation depths for concrete specimens.

Similar behavior was observed in the results of carbonation tests, as shown in Figure 5. For instance, carbonation depths of 10.2, 8.3, and 7.5 mm were measured for CB, CQ, and CG concrete specimens, respectively. A linear relationship with a R^2^ value of 0.94 was observed for these results. However, the addition of silica fume reduced the carbonation depths. For example, the specimens made with basalt, quartzite, and granite coarse aggregates with silica fume demonstrated carbonation depths of 8.2, 7, and 6.2 mm, respectively. R^2^ value of 0.98 for these results was noticed. 

The low resistance of the concrete specimens made with basalt aggregate may be due to the porous nature of the transition zone, which permitted the ingress of chloride ions and carbon dioxide. On the other hand, the transition zone along the granite and quartzite aggregate surfaces were denser compared to that of the basalt aggregate. Moreover, the addition of silica fume improved the overall microstructure of the cement composites, resulting in an enhanced resistance to chloride penetration and carbonation. Gesoglu et al. [22] produced self-compacting concrete using various mineral admixtures. The results of their study suggest that the chloride ion permeability decreased, resulting in improved durability of concrete. Similarly, the results of the work conducted by Song et al. [23] demonstrate that the relative diffusivity of bulk paste and ITZ decreased with the rise in silica fume content. Gonen and Yazicioglu [24] investigated the performance of concrete after the addition of mineral admixtures, silica fume, and fly ash. The depth of carbonation was lower for the concretes containing mineral admixtures.

**Figure 6:**
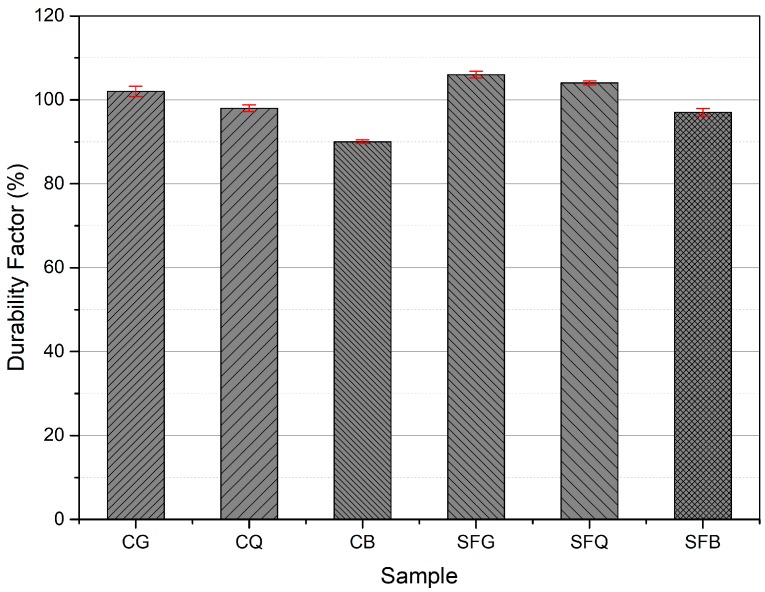
Durability factors for concrete specimens after freezing and thawing.

The durability factor was calculated from the results of freeze and thaw tests after 300 cycles, as shown in Figure 6. The specimens with basalt aggregates had the lowest values for the durability factor among all the specimens. For example, durability factors of 90%, 98%, and 102% were calculated for CB, CQ, and CG concrete specimens, respectively. These results presented a linear trend with R^2^ value of 0.96. Apart from this, the addition of silica fume improved the performance of concrete specimens against freezing and thawing. For instance, durability factors of 97%, 104%, and 106% were calculated for SFB, SFQ, and SFG specimens, respectively. A linear trend and R^2^ value of 0.90 was observed for these results. This is due to the fact that the addition of silica fume densified the microstructure of the cement composites, thus inhibiting the accumulation of freezing water inside the composites. This further improved the performance of the cement composites against freezing and thawing environments. Cwirzen and Penttala [25] reported that scaling decreased with increasing silica fume content. The results also indicate that damaging mechanisms initiate and accelerate within the transition zone by augmenting movement of the pore solution during freezing and thawing cycles. They established that the addition of silica fume appeared to densify the microstructure of the transition zone.

### 3.4. Scanning Electron Microscopy

**Figure 7:**
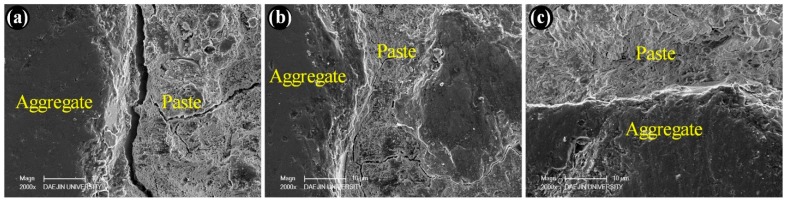
SEM micrographs of plain concrete made with (**a**) basalt, (**b**) quartzite, and (**c**) granite coarse aggregates.

The microstructure of the concrete specimens was investigated via scanning electron microscopy. Specimens were taken from the portion of the concrete specimens used for the compressive strength test. Figure 7 shows the SEM micrographs of the various specimens made without silica fume as a cement replacement material. Figure 7a–c represents the specimens containing basalt, quartzite, and granite coarse aggregates. The application of compression force induces cracking inside the specimens. These cracks preferentially initiate within the transition zone, which is the weakest region between the aggregate and the cement paste. It is obvious from the SEM images that the specimens made with basalt coarse aggregates showed a distinct crack along the aggregate surface. This means that the basalt aggregate did not provide a better interlocking performance with the cement paste. On the other hand, the SEM micrographs of the specimens made with coarse granite and quartzite aggregates demonstrate that cracks appeared away from the aggregate surface. This suggests that the near surface of these aggregates was stronger, so that the cracks did not initiate as close to the surface of the aggregates.

**Figure 8:**
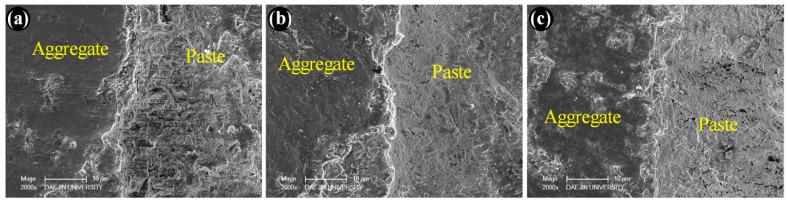
SEM micrographs of concrete containing silica fume and (**a**) basalt, (**b**) quartzite, and (**c**) granite coarse aggregates.

**Figure 9:**
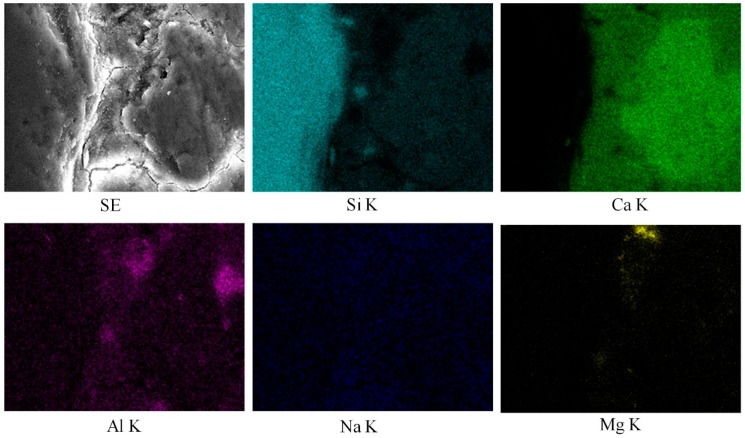
EDS mapping results of concrete specimens made with granite and OPC.

**Figure 10:**
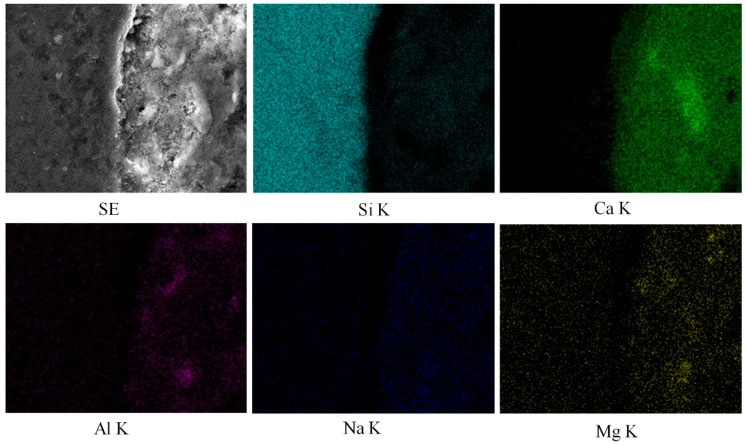
EDS mapping results of concrete specimens made with granite, OPC, and silica fume.

Figure 8 depicts the SEM micrographs of the specimens made with silica fume as a cement replacement material. Figure 8a–c represents the specimens containing basalt, quartzite, and granite coarse aggregates. Cracks are not visible near the aggregate surface. This is because the silica fume particles provided a better packing ability near the aggregate surface [6]. Additionally, silica fume particles caused a pozzolanic reaction, which consumed calcium hydroxide and densified the microstructure. This behavior is further demonstrated with the help of EDS mapping, as shown in Figure 9 and Figure 10. Mapping images show the abundance of each element in a selected area under observation [26]. Figure 9 and Figure 10 portray the mapping results for the CG and SFG specimens, respectively. It is important to note here that the selection of the specimens was made just to investigate the effect of silica fume on the microstructure. From these images, the pozzolanic behavior of silica fume can easily be perceived. 

## 4. Conclusions

This study used three kinds of coarse aggregates (basalt, quartzite, and granite) to make concrete specimens. Additionally, silica fume was used to replace cement at a replacement level of 10%. The specimens were analyzed in terms of compressive strength, ultrasonic pulse velocity, chloride penetration, carbonation, freeze–thaw resistance, and scanning electron microscopy. Based on the results, the following conclusions can be made.

1. Compressive strength increased with the addition of rougher coarse aggregates (i.e., granite and quartzite). On the other hand, the addition of silica fume resulted in an enhanced compressive strength for specimens containing basalt.
2. Pulse velocity test results reveal that comparable UPV values were obtained for the specimens made with different coarse aggregates.
3. An increased resistance to chloride penetration, carbonation, and freeze–thaw was observed for the specimens made with granite and quartzite compared to that made with basalt aggregate. However, the incorporation of silica fume significantly improved the performance of the concrete specimens.
4. The microstructures of the transition zones in the concrete specimens made with granite and quartzite coarse aggregates were denser in comparison to that made with basalt coarse aggregates. In the same way, the addition of silica fume improved the microstructure of all the specimens, irrespective of the aggregate types.

It can be concluded that the performance of the concrete can be improved with the addition of coarse aggregates with a rougher surface. Additionally, the incorporation of silica fume can enhance the performance of concrete, with smoother surface coarse aggregates.

This study did not consider the chemical interaction of the aggregates and the cement compounds.

## Figures and Tables

**Table 1:** Chemical composition and physical properties of OPC and silica fume.

Composition	Weight (%)	
	OPC	Silica Fume
SiO_2_	20.8	88.7
Al2O_3_	6.3	1.8
Fe2O_3_	3.2	1.8
CaO	62	1.5
MgO	3.3	0.8
SO_3_	2.2	0.1
Loss on ignition	1.3	1.1
Sp. Surface area (cm^2^/g)	3200	200,000

**Table 2:** Physical properties of coarse aggregates.

Aggregate	Sp. Gravity (SSD)	Absorption (%)	Fineness Modulus	Abrasion (%)
Quartzite (Q)	2.63	1.05	7.01	45
Basalt (B)	2.78	0.39	6.98	17
Granite (G)	2.56	0.85	7.02	38

**Table 3:** Chemical composition of coarse aggregates.

Composition	Weight (%)		
	Granite	Quartzite	Basalt
SiO_2_	75	75.02	48.78
Al_2_O_3_	15.24	6.25	11.02
Fe_2_O_3_	2.44	3.04	9.52
MnO	0.09	2.17	0.11
CaO	0.98	6.48	10.01
MgO	0.09	1.02	15.02
Na_2_O	1.57	1.2	2.54
K_2_O	2.49	1.97	0.55
TiO_2_	0.03	0.04	1
P_2_O_5_	0.23	0.01	0.23
